# Impact of rating and praise campaigns on local government environmental governance efficiency: Evidence from the campaign of establishment of national sanitary cities in China

**DOI:** 10.1371/journal.pone.0253703

**Published:** 2021-06-24

**Authors:** Genli Tang, Minghai Lin, Yilan Xu, Jinlin Li, Litai Chen

**Affiliations:** School of Public Policy and Administration, Chongqing University, Chongqing, China; China University of Mining and Technology, CHINA

## Abstract

**Background:**

Ecological and environmental protection is essential to achieving sustainable and high-quality development, which highlights the important role of environmental governance. In terms of the practical actions of environmental governance, the central government in China has carried out continuous rating and praise campaigns, and local governments have actively promoted this effort. However, the related performance consequences have not been empirically investigated. We aimed to verify whether this incentive policy can improve the efficiency of environmental governance and whether this governance method has long-term effects. In addition, we sought to identify mechanisms through which the policy can improve environmental governance.

**Method:**

We take the rating and praise campaign of the Establishment of National Sanitary Cities (EONSCs) as a quasi-natural experiment and use the panel data for 174 cities from 2004 to 2016 and the propensity score matching-difference in differences (PSM-DID) method to test the impact of rating and praise campaigns on environmental governance efficiency.

**Results:**

EONSCs campaign can improve the efficiency of environmental governance by 0.7595 (p<0.01), which is significant at the 1% level; the effects are clearly significant during the evaluation process and the year in which cities are named National Sanitary Cities (NSCs) but decrease annually thereafter. The EONSCs campaign has a significant promoting effect on public services provision, such as public infrastructure investment, public transportation and education.

**Conclusions:**

(1) The rating and praise campaigns can effectively improve the efficiency of environmental governance; (2) the incentive effect is distorted and is not a long-term effect; (3) the impact of the rating and praise campaign of EONSCs on the efficiency of environmental governance is mainly realized through the provision of corresponding public services that are closely related to environmental protection. The findings of this paper provide empirical support for the effectiveness of the central government’s rating and praise campaigns and could motivate local governments to actively participate in environmental governance. Moreover, the findings provide an important reference for further improving the rating and praise campaigns and the level of environmental governance.

## Introduction

The efficiency of environmental governance is a hot topic among scholars around the world [1, 2]. Existing studies on promoting environmental governance mainly focus on the large amount of work done by public organizations and actors in promoting environmental governance from a top-down perspective, considering the local government’s environmental governance efficiency based on the "one size fits all" and indicator downpressure assessment mode [3, 4], while little attention has been given to the voluntary participation of local governments in environmental governance. That is, there are few studies on the efficiency of environmental governance under the rating and praise mode in which local governments can choose whether to participate or not.

China’s unique political personnel system creates an ideal environment for researchers to investigate whether a rating and praise incentive policy improves the efficiency of environmental governance. After a long period of extensive growth in China, people have become increasingly aware of the importance of protecting the ecological environment [5, 6]. To this end, the central and local governments at all levels have gradually begun to improve the level of environmental governance, seeking to build an effective environmental governance system [7, 8]. In the specific implementation process, governments or responsible departments at all levels not only formulate environmental protection regulations and policies and increase investment in governance but also carry out various rating and praise campaigns to promote environmental governance [9, 10]. One of the most important measures is the selection of National Sanitary Cities (NSCs) [11, 12]. The rating and praise campaign for the Establishment of National Sanitary Cities (EONSCs) advocated by the National Patriotic Health Campaign Committee (NPHCC) has a long history [11–13]. The EONSCs campaign is an important means for the central government to encourage local governments to actively invest more human, material and financial resources into environmental governance.

The existing literature on the EONSCs campaign is not sufficiently in depth, focuses on how to create and manage NSCs, and lacks empirical research on the effect of the campaign [11, 14]. A few studies have used a single index to measure the environmental protection effect of the EONSCs, without exploring the transmission mechanisms and focusing on theoretical research [15]. How effective is the incentive mode of EONSCs? Does it improve the efficiency of environmental governance, and does it have long-term effects? What factors have helped EONSCs campaign to promote environmental governance efficiency, and what are the mechanisms behind this efficacy?

We adopt balanced panel data for 174 prefecture-level cities in China from 2004 to 2016 and use the propensity score matching-difference in differences (PSM-DID) method [16] to test the relationship between the local government’s EONSCs campaign and the efficiency of environmental governance and evaluate the effects of the rating and praise campaign. The contributions of this paper are as follows. (1) It is among the first attempts to study the efficiency of environmental governance from a new perspective—local governments’ voluntary participation in the EONSCs campaign, which is different from the previous top-down perspective, expanding the research content of environmental governance and enriching the literature in this field. (2) It empirically tests the direct and dynamic effects of the rating and praise from the local government’s EONSCs campaign and explores the mechanisms behind the rating and praise mode, making up for the lack of empirical research on the effect of the campaign in the previous literature and providing direct experiential support for improving the government’s rating and praise campaigns in the future. (3) It utilizes a scientific assessment method—the improved slack-based measure (SBM) model [17] to build a series of indicators to measure the efficiency of local environmental governance, which makes up for the limitation of using a single indicator to measure the environmental governance efficiency in existing research, and adopts the PSM-DID method of quasi-natural experiments to test the effects of the EONSCs campaign.

The remainder of the paper is structured as follows. Section 2 presents the data and methods used in our empirical analyses and offers descriptive statistics of the main variables. Section 3 reports our research results. Section 4 discusses the theoretical contributions and practical implications of our findings on the environmental governance effectiveness of rating and praise campaigns. Section 5 concludes.

## Materials and methods

### Samples and data sources

From 1990 to 2008, the NSCs were named sporadically every year, and no programmed standardized process was formed. There are a few cases from the period, but they are not sufficient for analysis. After 2008, the NPHCC began to publish the list naming sanitary cities (Table 1) for a three-year cycle in the form of a summary. This approach is more scientific, and it not only promotes the local governments’ enthusiasm for the EONSCs but also conforms to the actual evaluation process. The samples were divided into an experimental group and a control group. All NSCs within the period were classified into the experimental group. Cities that did not participate in the EONSCs or that participated in the EONSCs but were not named NSCs were the control group. Due to the differences in the evaluation criteria between the first two cycles and the third cycle after 2008, we mainly select the NSCs from the first cycle (2009–2011 cycle) for testing and the second cycle (2012–2014 cycle) for further robustness testing.

**Table 1:** The NSCs in the first three cycles published by the NPHCC after 2008.

No.	Cycle	City level	NSCs
1	2009–2011	Prefecture-level city	Jincheng, Ordos, Baotou, Yichun, Zhoushan, Tongling, Dezhou, Linyi, Anyang, Shiyan, Heyuan, Meizhou, Nanning, Guangan, Guiyang, Kunming, Yuxi, Xianyang, Weinan, Jinchang
2	2012–2014	Prefecture-level city	Wuhan, Heihe, Suqian, Yancheng, Xuzhou, Wenzhou, Lishui, Taizhou, Ji’an, Pingdingshan, Xinyang, Kaifeng, Xianning, Xiangyang, Loudi, Yiyang, Yongzhou, Chenzhou, Zhanjiang, Yunfu, Baise, Liuzhou, Deyang, Guangyuan, Pu’er, Lijiang, Qujing, Lhasa, Hanzhong, Ankang, Tongchuan, Yulin
3	2015–2017	Prefecture-level city	Xinzhou, Tongliao, Wulanchabu, Panjin, Meihekou, Lianyungang, Xuancheng, Jining, Hebi, Luohe, Nanyang, Zhumadian, Huangshi, E’zhou, Jingmen, Jingzhou, Huanggang, Shaoyang, Yangjiang, Qingyuan, Haikou, Yibin, Bazhong, Liupanshui, Anshun, Yan’an, Shizuishan, Wu Zhong

Source: compiled by the author.

The time at which the sample cities began to be impacted by the EONSCs campaign can be determined according to the declaration procedure and the actual practice of establishing an NSC. According to the Measures for the Assessment, Naming, Supervision and Management of the NSCs, the assessment cycle for an NSC was revised from two years to three years. The first quarter of each year is the declaration period, the next period is the evaluation period, and centralized publicity and naming as a sanitary city occur in the fourth quarter of the third year. The declared sanitary cities must be named provincial-level sanitary cities for more than two years. Therefore, declared cities can only apply to the provincial-level Patriotic Health Campaign Committee (PHCC) if they meet the basic conditions. Under the guidance of the provincial-level PHCC, only after all the indicators have met the requirements of the NSCs standards can a city be recommended for consideration by the NPHCC. The second step is review. The rating team under the NSCs Rating Commission conducts secret investigations, technical evaluations and social announcements on the applying cities. At the end of the third year or the beginning of the fourth year, the declared cities that have met the NSCs standards will be named centrally, and then the supervision and management of the NSCs will be reviewed every three years. Thus, recognition by the EONSCs campaign is not an overnight achievement.

Unlike the situation in which most policy effects are mainly reflected "after the implementation of the policy", the environmental governance level of cities participating in the EONSCs changes and gradually improves before a city is named an NSC [12]. Generally, the effects are achieved in the early stages of the EONSCs, but they are not obvious. The effects are most obvious in the middle and later stages of the EONSCs, especially in the sprint period and in the year cities are named NSCs [15].

Based on the actual practice of many evaluated NSCs, the 1–2 year period before the declared cities were officially named NSCs can be taken as the period for investigating policy impact. In summary, based on the NSCs of the first cycle, we identify the period from 2004 to 2007 as the base period of EONSCs and the period from 2008 to 2013 as the period for investigating the effect of EONSCs implementation. For the NSCs of the second cycle, we identify the period from 2007 to 2010 as the control base period of EONSCs and the period from 2011 to 2016 to investigate the effect of EONSCs implementation. Therefore, the period of study of all samples is from 2004 to 2016. In 2004, 283 prefecture-level cities were identified in the China Urban Statistical Yearbook. To maintain the consistency of the data, the number of cities counted in 2004 was used, and the newly established cities in subsequent years were not included. According to statistics released on the official website of the National Health Commission, by February 2019, the total number of NSCs was 159, of which 52 prefecture-level cities met the requirements of this study (in the first two cycles after 2008). Among them, the data for Lhasa are missing, so it is not included in the study. The remaining 124 prefecture-level cities were not named NSCs. Among them, the administrative level of a prefecture-level city was revoked for Chaohu city in 2011, so it is not included in the study. After sorting, 51 NSCs were included in the experimental group, and 123 non-NSCs were included in the control group. The data are derived from multiple sources. The data on environmental governance are collected from calculations by constructing an index system. Data about the EONSCs are gleaned from the official websites of the National Health Commission. Data on urban attributes are from the China City Statistical Yearbook.

### Model specifications and statistical test

The difference-in-differences (DID) model [18] is often used to control the difference between the experimental group and the control group before and after the policy’s implementation, and effectively identify the net policy effect. The DID method can largely avoid endogeneity problems by solving the problems of missing variables and selection bias to a certain extent. The cities participating in the EONSCs campaign are random. Compared with the traditional method, when the parallel trend assumption is satisfied, that is, in the absence of the EONSCs campaign, the environmental governance efficiency of the two groups follows the same trend over time, the DID method can estimate the policy effect more accurately [19]. The DID method takes the control group, which is not affected by the policy, as the counterfactual reference of the treatment group, which is affected by the policy. The difference in average outcome in the experimental group before and after the implementation of the EONSCs campaign minus the difference in average outcome in the control group before and after the implementation of the EONSCs campaign cleanly evaluates the treatment effect of the policy. Therefore, we also adopt the DID method for our evaluation.

The DID method can determine the "policy treatment effect" and solve endogeneity problems, but it cannot effectively eliminate the existence of selection bias. To reduce the potential selection bias as much as possible, it is essential that the matches between the control group and the experimental group include all aspects to support causal inference. We adopt propensity score matching (PSM) [20] to address possible selection bias.

Given the advantages of PSM and DID, we adopt the PSM-DID method to effectively overcome the problems of endogeneity and selection bias to more accurately estimate the effects of EONSCs campaign on urban environmental governance efficiency. Based on the above analysis, we set the benchmark regression model as follows:

$${\text{Effect}}_{\text{it}}=\alpha_{0}+\alpha_{1}{\text{treated}}_{\text{it}}\times {\text{time}}_{\text{it}}+{\alpha \text{X}}_{\text{it}}+\mu_{\text{i}}+\vartheta_{\text{t}}+\varepsilon_{\text{it}}$$ (1)

where i and t represent city i and year t, respectively. Effect_it_ is the behavioral effects of the EONSCs, referring to the environmental governance efficiency of the ith city in year t. The variable treated_it_ refers to the virtual variable of groups: treated_it_ = 1 denotes the experimental group; treated_it_ = 0 refers to the control group. The variable time_it_ represents a dummy variable for the year: time_it_ = 1 refers to the investigation period for the effects of the EONSCs, while time_it_ = 0 refers to the base period for those who have not carried out the EONSCs or before the EONSCs campaign becomes effective. The interaction term of two dummy variables, treated_it_ × time_it_, is an independent variable represented by EONSCs. The coefficient α_1_ of treated_it_ × time_it_ in Formula (1) refers to the net effect of the EONSCs campaign on urban environmental governance efficiency. X_it_ represents a set of control variables, μ_i_ represents city fixed effects, ϑ_t_ represents year fixed effects, and ε_it_ is the random disturbance term.

#### Dependent variable

Environmental governance efficiency describes the actual effects of EONSCs campaign in environmental governance, including reducing pollution emissions, achieving emission standards, reducing energy consumption, and improving the living environment. The governments’ environmental governance efforts and effects are difficult to directly measure. To reflect the improvement of environmental governance quality and avoid one-sidedness of the result indicators, we draw on the practices of Yu et al. [21] and Wang et al. [22] and use DEA, which is widely used in efficiency evaluation research to measure local governments’ environmental governance efficiency.

To solve the problem of slackness, such as undesired output that the traditional DEA model cannot solve, we adopt the improved SBM model in DEA and construct 3 input indicators and 6 output indicators, which can comprehensively evaluate the policy effect of EONSCs campaign. According to the analytical perspective of environmental economics [23], the input elements of environmental governance generally include capital, manpower and technology. Therefore, we select government environmental protection input, environmental management practitioners and science and technology input as input indicators [24]. It is commonly understood that environmental governance requires government investment and personnel. Government expenditure on science and technology provides support for technological innovation and environmental scientific research, which can guide the agglomeration of regional ecological elements and the transformation and upgrading of industrial structure. Output indicators are divided into expected output and unexpected output. With references to previous research, the comprehensive utilization rate of industrial solid waste, sewage treatment rate, and per capita green area are selected as expected output indicators [25]; wastewater discharge, industrial smoke (dust) emissions, and sulfur dioxide emissions are selected as unexpected output indicators [26].

#### Control variables

With reference to existing research [27–29], we select a series of control variables. The measurement indicators of the economic development level are relatively mature. Most studies express this as per capita gross domestic product (GDP), which reflects the economic development status of a certain region. Green public infrastructure investment and construction can improve the environmental governance effect [30, 31], and then public infrastructure investment is represented by the ratio of actual investment in fixed assets to the total population. The degree of industrialization is measured by the proportion of secondary industry in the gross domestic product. The emission of exhaust from a large number of private cars has a certain impact on environmental governance, and the promotion of public transportation is conducive to the efficiency of environmental governance [32]. Thus, the number of publicly operated vehicles per capita is expressed by the ratio of the number of publicly operated vehicles to the total population. The education level is indicated by the ratio of the number of ordinary middle school students to the total population. The description and measurement of related variables and the descriptive statistics of the main variables are shown in Table 2.

**Table 2:** Main variables and descriptive statistics.

Variable	Variable symbol	Variable definition	Observations	Mean	SD	Max	Min
Environmental governance efficiency	Ege	Environmental governance efficiency after measurement	1430	0.931	2.445	53.893	0.009
Economic development level	Lnpgdp	Logarithm of per capita GDP	1430	10.138	0.726	12.690	7.520
Public infrastructure investment	Infra	The ratio of actual fixed asset investment to total population	1430	2.226	2.213	24.159	0.051
Industrialization	Lnind	Logarithm of the ratio of secondary industry to GDP	1430	3.861	0.307	4.504	2.086
Foreign direct investment	Lnfdi	Logarithm of the actual amount of foreign direct investment	1430	8.096	1.823	12.450	1.070
Buses / electric trolleybuses	Pbus	The ratio of the number of buses / electric trolleybuses to the total population	1430	6.391	1.009	9.550	3.700
Education level	Lnedu	Logarithm of the ratio of the number of ordinary middle school students to the total population	1430	6.429	0.310	7.458	4.502

## Results

### Benchmark model test (DID)

#### Parallel trend test

In this paper, the DID method is used to estimate the impact of rating and praise campaigns on the environmental governance efficiency of local governments. However, the premise of establishing the DID method is that the development trend of the environmental governance efficiency of the treatment and control groups should be parallel before the implementation of the EONSCs campaign. Following the event study method [33], we carry out a parallel trend test. The test model is constructed as follows:

$${\text{Effect}}_{\text{it}}=\alpha_{0}+\sum_{k=-4}^{5}\delta_{\text{k}}{\text{treated}}_{\text{i}}\times {\text{year}}_{\text{k}}+{\alpha \text{X}}_{\text{it}}+\mu_{\text{i}}+\vartheta_{\text{t}}+\varepsilon_{\text{it}}$$ (2)

The year_k_ denotes the year dummy variable, the value of the observed year is 1, and the value of other years is 0. We define k = -4 as the base year. The coefficient δ_k_ of treated_i_ × year_k_ measures the difference between the treatment group and the control group in phase k. Other variables are consistent with the baseline model. If the δ_k_ statistic results before the implementation of the EONSCs campaign are not significant, then the parallel trend hypothesis is satisfied.

**Fig 1:**
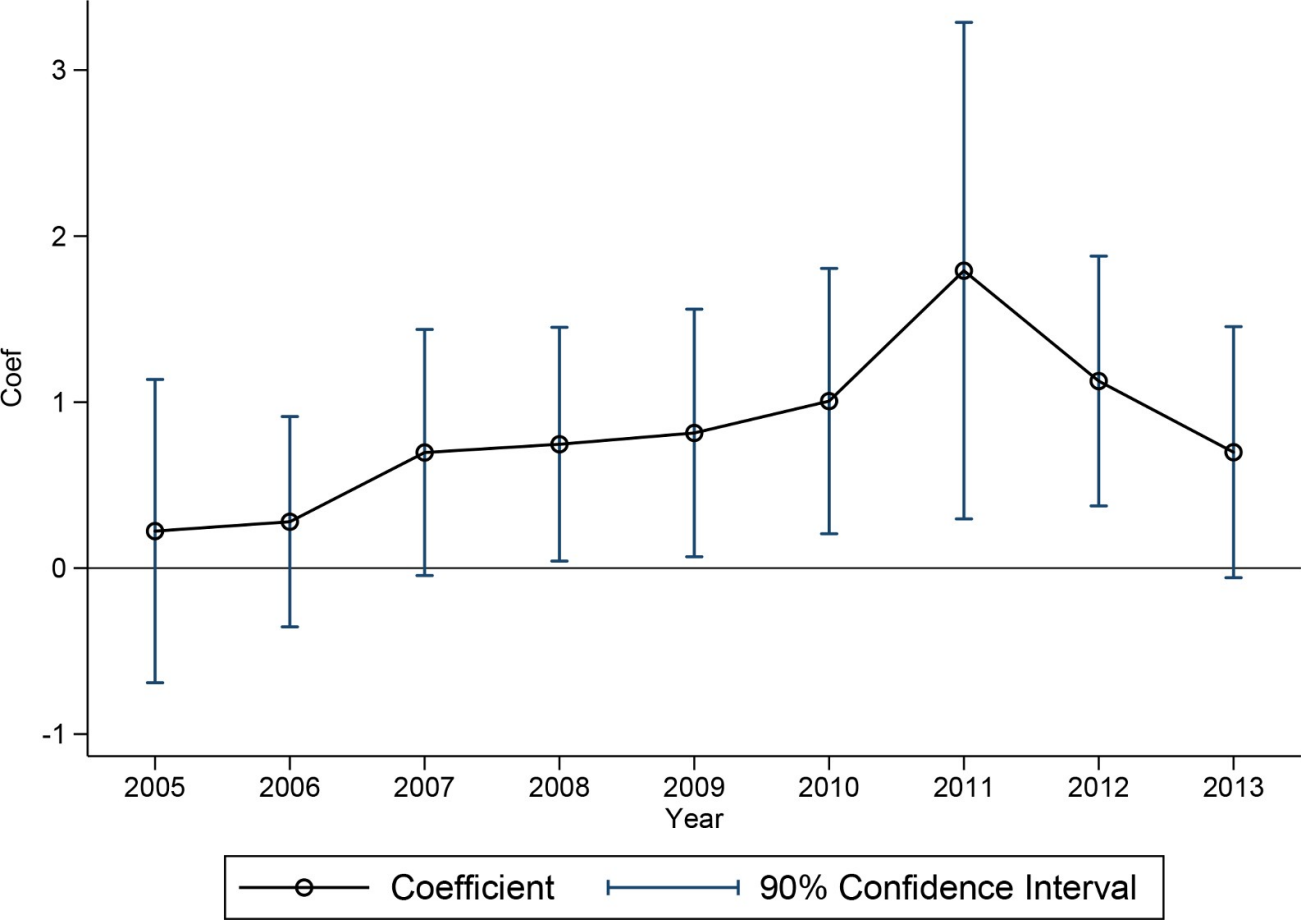
Parallel trend test coefficient diagram.

We present the environmental governance effects of the EONSCs campaign in different years by means of visual graphics (Fig 1). Fig 1 shows that the estimated coefficients fluctuate around 0 before the implementation of the EONSCs campaign, while the coefficients are significantly positive in the year after the implementation of the EONSCs campaign and in subsequent years. This trend indicates that the difference between the experimental group and the control group before the implementation of the EONSCs campaign is not obvious and that the groups can be compared, meeting the premise of a parallel trend.

Fig 1 also shows that the estimated coefficients show a decreasing trend year by year after 2011. This finding shows that the marginal effect of the EONSCs campaign decreases over time. That is, the environmental governance efficiency of the second and third years after the selection was announced is declining. This trend shows that EONSCs campaign has an immediate effect and then exhibits a downward trend annually. The rating and praise campaign has an incentive distortion effect and lacks a long-term impact. As a practical observation, the main reason the EONSCs campaign fails to achieve the expected long-term effect is that the honorary title of an NSC is understood as a "life tenure system", and the lack of supervisory mechanisms from higher-level departments means that the provision of supporting public services in the NSCs lags or is terminated, and the local governments slowly cease to devote energy and public resources to the campaign.

#### Baseline regression results

Based on the benchmark model (1), we use the DID method to test the policy effect of EONSCs. The specific results are shown in Table 3. Column 1 is the regression result before adding control variables, and column 2 is the estimation result after adding control variables. Regardless of whether control variables are added, the coefficient of the interaction term treated × time is significantly positive, which indicates that EONSCs campaign significantly improves the efficiency of environmental governance and the quality of the ecological environment of the residents. According to column 1, EONSCs campaign (treated × time) increases the environmental governance efficiency by 0.7255, which is significant at the level of 1%, indicating that the EONSCs campaign has a significant impact on the level of environmental governance; according to column 2, when the control variables are added, the EONSCs campaign increases the efficiency of environmental governance by 0.7151, indicating that the EONSCs campaign has a significant role in promoting the level of environmental governance.

**Table 3:** Regression results of the benchmark model (DID).

	Ege (1)	Ege (2)
EONSCs	0.7255***	0.7151***
	(2.87)	(2.89)
Year fixed effects	Yes	Yes
City fixed effects	Yes	Yes
Control variables	No	Yes
_cons	1.4850***	12.1991
	(4.12)	(1.22)
N	1430	1430
R^2^	0.0186	0.0291

Note: t-statistics in parentheses;

*** represents the 1% significance level.

### Robustness tests

#### Counterfactual test

To further test the robustness of our results, we refer to the existing research [34, 35] and conduct a counterfactual test by changing the implementation time of EONSCs campaign. In addition to the policy change of the implementation of EONSCs campaign, some other policies or random factors may also lead to differences in the efficiency of local environmental governance. However, such differences are not related to the implementation of EONSCs campaign, which ultimately invalidates the previous conclusions. To eliminate the influence of such factors, we assume that the year of the implementation of EONSCs campaign in each city is uniformly two or three years ahead. If the coefficient for the interaction term treated × time becomes significantly positive at this time, it indicates that local environmental governance efficiency is likely to come from other policy changes or random factors rather than the implementation of EONSCs campaign. If the coefficient for the interaction term treated × time is not significantly positive at this time, it indicates that the efficiency change of local environmental governance comes from the implementation of the EONSCs campaign.

Table 4 shows the results of the placebo test for our DID model. All results show that the coefficients for the interaction term treated × time are not significant, implying that the efficiency change of local environmental governance is not caused by other factors but by the implementation of the EONSCs campaign. Therefore, the DID estimation results in Table 3 are robust.

**Table 4:** Placebo test results.

	Ege (1)	Ege (2)
EONSCs.adv2	0.1410	0.0541
	(0.51)	(0.18)
EONSCs.adv3	0.2687	0.2234
	(0.47)	(0.41)
Year fixed effects	Yes	Yes
City fixed effects	Yes	Yes
Control variables	No	Yes
_cons	1.4850***	12.3606
	(4.12)	(1.24)
N	1430	1430
R^2^	0.0193	0.0296

Note: t-statistics in parentheses;

*** represents the 1% significance level.

#### PSM-DID inspection

The reliability of PSM depends on whether the assumption of "negligibility" is met. Therefore, a balance test must be conducted before using the PSM-DID method. According to Rosenbaum and Rubin [36], if the absolute value of the standard deviation after matching is less than 20%, then the matching effect requirements are met. The balance test results (Table 5) indicate that the matching variables and matching methods selected in this paper are appropriate.

**Table 5:** Balance test results.

Variable	Unmatched	Mean		Bias (%)	Reduct |bias| (%)	T-test	
	Matched	Treated	Control			t	p>|t|
Lnpgdp	Unmatched	10.546	10.072	66.5	92.9	8.79	0.000
	Matched	10.519	10.485	4.7		0.47	0.363
Infra	Unmatched	3.322	2.0478	45.0	96.2	7.70	0.000
	Matched	3.094	3.0453	1.7		0.17	0.862
Lnind	Unmatched	3.959	3.8445	40.6	85.6	4.93	0.000
	Matched	3.964	3.9474	5.8		0.65	0.516
Lnfdi	Unmatched	8.550	8.0225	30.2	76.6	3.81	0.000
	Matched	8.540	8.4165	7.1		0.72	0.473
Pbus	Unmatched	6.377	6.3936	-1.7	-230.9	-0.22	0.825
	Matched	6.403	6.3468	5.5		0.54	0.588
Lnedu	Unmatched	6.497	6.4183	24.4	93.6	3.32	0.001
	Matched	6.495	6.5001	-1.6		-0.15	0.878

After propensity score matching and the balance test passed, we re-estimate the environmental governance effect of the EONSCs campaign. The results of the PSM-DID robustness test in Table 6 show that there is a highly significant positive correlation between the EONSCs campaign and the efficiency of environmental governance after matching. This finding indicates that the EONSCs campaign can promote the improvement of environmental governance. After matching, the EONSCs campaign still improves the efficiency of environmental governance by 0.7595, which confirms that under the rating and praise mode, the cities participating in the EONSCs have higher environmental governance efficiency.

**Table 6:** The results of the robustness test for the PSM-DID analysis.

	Ege (1)	Ege (2)
EONSCs	0.7595***	0.7154***
	(3.00)	(2.83)
Year fixed effects	Yes	Yes
City fixed effects	Yes	Yes
Control variables	No	Yes
_cons	1.4997***	12.9250
	(4.11)	(1.28)
N	1416	1416
R^2^	0.0199	0.0306

Note: t-statistics in parentheses;

*** represents the 1% significance level.

#### Using an alternative sample

To ensure the stability of the empirical results, we use data for the second cycle of the NSCs. The PSM-DID method is again used for the empirical analysis after the DID parallel test, counterfactual test and PSM balance test are passed. In essence, a quasi-natural experiment similar to the NSCs selection campaign in the first cycle is repeated using the new sample data, and the measurement results (Table 7) show that the net change in environmental governance level in the NSCs before and after the EONSCs campaign reaches 0.1380 and remains highly significant at the 1% level. The empirical results once again show that EONSCs campaign can improve the level of local governments’ environmental governance, which is still valid under the new experimental scenario.

**Table 7:** The results of the robustness test for PSM-DID using data in the second cycle.

	Ege (1)	Ege (2)
EONSCs	0.1380***	0.1126***
	(3.51)	(3.04)
Year fixed effects	Yes	Yes
City fixed effects	Yes	Yes
Control variables	No	Yes
_cons	3.1043***	-0.7025
	(117.66)	(-0.79)
N	1507	1507
R^2^	0.1498	0.2022

Note: t-statistics in parentheses;

*** represents the 1% significance level.

### Screening of influence mechanisms

Considering all the control variables that affect the efficiency of environmental governance, we use EONSCs (treated × time) to regress all the control variables to identify which transmission channels are responsible for the failure of EONSCs campaign to effectively promote improvements in environmental governance efficiency over the long term. The results are shown in Table 8.

**Table 8:** Mechanisms screening of the impact of the EONSCs campaign on environmental governance efficiency.

	Lnpgdp	Infra	Lnind	Lnfdi	Pbus	Lnedu
EONSCs	-0.0938*	1.1337*	-0.0902***	0.0355	0.0620**	0.1167*
	(-1.84)	(1.81)	(-3.22)	(0.18)	(2.37)	(1.84)
Year fixed effects	Yes	Yes	Yes	Yes	Yes	Yes
City fixed effects	Yes	Yes	Yes	Yes	Yes	Yes
_cons	9.4606***	0.7319***	3.8462***	7.2291***	6.4181***	6.5265***
	(506.75)	(7.43)	(274.24)	(89.67)	(281.67)	(431.06)
N	1430	1430	1430	1430	1430	1430
R^2^	0.8793	0.5817	0.1193	0.3200	0.0186	0.1206

Note: t-statistics in parentheses; ***, ** and * represent the 1%, 5% and 10% significance levels, respectively.

According to Table 8, the EONSCs campaign has different degrees of restraint on the level of economic development and industrialization and has a significant promoting effect on investment in urban public infrastructure construction, the number of buses/electric trolleybuses per capita, and the level of education. The effect of foreign direct investment is positive but not significant. These results show that the impact of the rating and praise campaign of EONSCs on the efficiency of environmental governance is mainly realized through public services provision, such as public infrastructure investment, public transportation and education. The implementation of EONSCs will inevitably have a certain impact on economic development. Most local governments sacrifice a certain amount of economic development in the short term to reach sanitary standards. Once the evaluation criteria are met or the honorary title is achieved, the focus turns to GDP development. Similarly, during the period of developing the EONSCs, to some extent, manufacturing industries that produce harmful substances or pollute air and water will be negatively affected, which will affect the development of industrialization in the short term. The NSCs standards require that the jurisdiction has not experienced a major accident causing environmental pollution or ecological damage over the past three years, that its ambient air quality index or air pollution index has not exceeded 100 in ≥ 300 days, and that its annual average value of major ambient air pollutants meets the second level of the national Ambient Air Quality Standard. Therefore, the EONSCs campaign has a certain inhibitory effect on air pollution caused by industrial smoke and dust. Through efforts to improve urban appearance and environmental sanitation, environmental protection, sanitation in key locations, food and drinking water safety, public health and medical services, the EONSCs campaign may attract a certain amount of foreign direct investment. Then, according to real-world observations, before the selection of NSCs, the participating city governments will improve the supporting public services of environmental protection and improve the quality of public services provision related to environmental protection. For example, cities may add a number of dedicated garbage depots and bins (barrels), improve drainage facilities, prohibit open drainage ditches, strengthen education efforts on environmental protection and publicize scientific information on environmental protection. In short, the EONSCs campaign can significantly promote the efficiency of local governments’ environmental governance in the short term, mainly through the improvement of a series of public services provisions closely related to environmental protection.

## Discussion

There are extensive political promotion tournaments in the economic field, and scholars from various countries have conducted in-depth research to explain the incentive mechanism of China’s officials and economic growth [37, 38]. On the other hand, officials are also encouraged to build noneconomic fields through competition. These incentive mechanisms can also be classified into a political promotion tournament system, and this type of tournament is commonly referred to as the rating and praise mode [14, 39, 40]. Compared with a large amount of research in the economic field of political promotion tournaments, academic circles have paid little attention to political standards competitions in noneconomic fields [11, 12, 14]. Apart from the characteristic that the competition takes place in noneconomic fields, this competition mode is also different from the "one size fits all" indicator-based downward pressure evaluation system. This mode can give full consideration to the initiative of local governments and whether they voluntarily choose to participate in political standards competitions in noneconomic fields, namely, rating and praise campaigns [12, 14]. In recent years, there have been an increasing number of political standards competitions around the honorary titles of cities in China, such as the selection of NSCs, National Civilized Cities (NCCs) and National Garden Cities (NGCs). There are many rating and praise campaigns in noneconomic fields, and local governments and their officials are willing to participate in such competitive rating and election campaigns [11, 12, 14]. In addition to political officials’ promotion considerations, the rating and praise mode not only has the advantage of mobilizing and concentrating human and financial resources in a short period of time but will also provide corresponding rewards for successful ratings and elections [11]. In addition, rating and praise campaigns can play a specific role in overall economic and social development. Therefore, we focus on the expected effect of rating and praise.

As a typical representative of the rating and praise mode in China, the EONSCs campaign has a long history. This campaign can be traced back to October 1989, when the NPHCC issued the Notice of the National Patriotic Health Campaign Committee on the Selection of NSCs, which initiated the national EONSCs campaign. At the same time, the NSCs Standards and the Assessment, Naming, Supervision and Management of the NSCs were also promulgated. Since then, the standards and measures have been revised many times, and the index system has become increasingly elaborate and complex. These indicators include so-called "hard indicators" that are relatively easy to objectively measure and "soft indicators" that rely on the subjective judgment of members of the investigative teams. On the one hand, these indicators show the work requirements and expectations of superior institutions in environmental governance. On the other hand, they also become competitive indicators of promotion tournaments in a noneconomic field.

Under the rating and praise mode, some cities did not carry out EONSCs campaign, while some cities actively participated in EONSCs campaign [11, 14]. Based on their actual conditions, these cities made their own decisions to respond to the initiatives of the central government [14, 41]. To conscientiously implement the Decision of the State Council on Strengthening the Patriotic Health Work, the local governments that are participating in the EONSCs will put patriotic sanitary work on their agendas, include it in their social and economic development planning, and formulate relevant regulatory documents [9]. At the same time, local cadres are attaching great importance to the establishment of organizational structures for the local PHCC: multiple departments participate, and generally, the city’s chief officer (or deputy) acts as the general commander to coordinate the actions of these different departments [14]. The member units of the local PHCC have clear rights and responsibilities, and it has a special financial budget, formulates patriotic sanitary work plans and annual plans, and creates a patriotic sanitary campaign in which all departments, units and broad citizenry actively participate [11, 14].

With the continuous development of China’s economy and society, the construction of an ecological civilization has become more prominent in the cause of Chinese socialism, and ecological environmental protection has become a hot topic of discussion. Environmental problems are a typical dilemma in public governance. Governments can guide society to conduct environmental governance through institutional arrangements [42, 43], such as environmental policy tools [44, 45], financial arrangements [46, 47], and environmental protection assessments and inspections [48, 49]. Ecological environmental protection is inseparable from the intervention and governance of public power departments. EONSCs campaign, as a public governance campaign in which local government officials follow their own wishes to conduct environmental governance, not only involves the evaluation and promotion of local officials but also truly concerns local environmental governance. EONSCs campaign is expected to produce a certain green effect. Therefore, to achieve sustainable, green and high-quality development in China, the EONSCs campaign is essential. Studies have shown that carrying out EONSCs campaign can promote an increase in green space and reduce industrial sulfur dioxide and wastewater discharge [11, 12]. From the perspective of evaluation indicators, the NSCs evaluation indicators include urban green coverage, domestic waste treatment rate, pollutant index reduction, number of days with good air, and water quality. Theoretically speaking, carrying out EONSCs campaign can also improve the level of city appearance and environmental sanitation so that urban household garbage and sewage can be treated in a centralized and harmless manner, and the urban green environment will be cleaner. Cracking down on straw burning can help the air pollution index reach the national standard and reduce the occurrence of major environmental pollution and accidents causing ecological damage. Carrying out the EONSCs campaign can further improve the supervision mechanism for the processes ensuring drinking water safety, implementing quantitative grading for sanitation supervision in key public places, and avoiding major water pollution accidents. Therefore, the EONSCs campaign can improve the quality of the ecological environment.

In the era of China’s reform and development, campaign-style governance at the local level is manifested in the selection of NSCs, environmental protection, traffic control and other campaign-style law enforcement activities [50]. Campaign-style governance improves the efficiency of governance by quickly mobilizing administrative resources and public power to reconstruct the social order in a short period of time [51, 52]. However, campaign-style governance does not have a long-term sustainable effect in terms of achieving its goals, it is easy for cities to regress once the goal is achieved, and it distorts the effectiveness of governance. The EONSCs campaign, as a governance action in which the local government plays an active role, inevitably leads local governments to invest a large amount of administrative resources to achieve an honorary title. From the perspective of practical operations, to achieve the title of an NSC, local governments vigorously and rapidly promote the realization of short-term assessment standards with rigid measures. This "hard index" and "military order" type of political mobilization stimulates local governments to invest many human, material and financial resources into the EONSCs. Although such emergency measures taken to achieve NSCs status have an obvious positive effect on environmental governance in the short term [11], the substantial effect is greatly compromised, and this type of governance will not with a long-term, stable and lasting effect [53]. It is very difficult to establish a long-term mechanism to improve the quality of environmental governance. Therefore, under the rating and praise mode, the EONSCs campaign has the effect of improving the quality of environmental governance, but it does not have a long-term effect.

The intention of local governments to participate in the rating and praise campaign is obvious. Regardless of whether local officials will be promoted as a result, they should first consider how to obtain political capital, that is, how to achieve the intended effect of environmental governance improvement through the rating and praise campaigns, to further consider the stability of their position and the potential for promotion [14]. As the rating and praise campaign of active participation of governments, a large amount of public resources must be invested to improve the quality of environmental governance [12]. In the practice of rating and praise campaigns, local governments seldom purchase public services or adopt the public-private partnership (PPP) mode to achieve their goals but more often realize the effect of rating and praise campaigns through the public service provision of the public sectors themselves [15]. Public service provision means that governments provide services to society and the public through public power intervention or public resource input [54]. In terms of environmental governance improvement, a large number of studies have proven that public service provision has a certain effect on the quality of environmental governance [55, 56]. In the process of EONSCs campaign, the investment in urban public infrastructure, such as the construction of garbage disposal points, garbage houses and dustbins, can reduce the area of environmental pollution; large investments in buses and electric trolleybuses can reduce air pollution caused by the use of private cars. At the same time, strengthening education obligations and raising people’s awareness of environmental protection can also promote the efficiency of environmental governance. Accordingly, under the rating and praise mode, the EONSCs campaign improves the quality of environmental governance through public service provision.

## Conclusions

The national campaign for the EONSCs is an innovative means to improve the level of environmental governance [11], and it can effectively leverage the enthusiasm of local governments to participate in environmental protection [14]; however, accurately identifying the policy effect of the rating and praise campaign of the EONSCs is a common concern. Based on the quasi-natural experiment of the NSCs selection, we use balanced panel data of 174 prefecture-level cities in China from 2004 to 2016 and the PSM-DID method to empirically test the relationship between rating and praise campaigns and environmental governance efficiency. We further analyze whether the EONSCs campaign has long-term effects, carry out the relevant robustness tests and screen for the relevant mechanisms. Our results reveal that (1) carrying out the EONSCs campaign can effectively promote improvements in environmental governance efficiency. From this perspective, continuing to deepen the rating and praise campaign can be an important way to improve the urban environmental protection level. (2) According to the results of the dynamic effect test, the EONSCs campaign has significant effects on improvements in the environmental governance efficiency before the announcement of the EONSCs rating results and in the year when the focal city is named an NSC, but its effects are not always significant thereafter, and it has no long-term effect. This finding provides direction for improvements to the rating and praise mode and to the mechanisms for the improvement of environmental governance. (3) The results of the mechanisms screening show that under the rating and praise mode, the improvement of public services provision, such as supporting public infrastructure, public transportation and education in the EONSCs process, has promoted the improvement of environmental governance efficiency.

## Supporting information

S1 Dataset Analytical dataset. (XLSX)
